# 2065. Efficacy of D-peptide-conjugated Antisense Compounds Against *Pseudomonas aeruginosa*

**DOI:** 10.1093/ofid/ofad500.135

**Published:** 2023-11-27

**Authors:** Rachelle E Koch, Dina A Moustafa, Christine A Pybus, Joanna B Goldberg, David E Greenberg

**Affiliations:** University of Texas Southwestern Medical Center, Dallas, TX; Emory University School of Medicine, Atlanta, Georgia; UT Southwestern Medical Center, Dallas, TX; Emory University School of Medicine, Atlanta, Georgia; UT Southwestern, Dallas, Texas

## Abstract

**Background:**

*Pseudomonas aeruginosa* (PA) causes significant morbidity and mortality in immunocompromised hosts and those with cystic fibrosis. Peptide-conjugated phosphorodiamidate morpholino oligomers (PPMOs) are antisense compounds designed to target specific genes and prevent translation. PPMOs with L-isomer peptides (L-PPMOs) were previously shown to have *in vitro* and *in vivo* activity. However, L-PPMOs are vulnerable to degradation by host proteases. We hypothesize that PPMOs with a D-isomer peptide (D-PPMOs) may be a more stable and potent alternative. We aimed to characterize the activity of L- and D-isomers in PA strains using planktonic and biofilm assays as well as an infected mouse model.

**Methods:**

All L- and D-PPMOs were synthesized by Sarepta Therapeutics, Inc. We tested L- and D-isomers of the (RXR)4XB (X for 6-aminohexanoic acid and B for β-Ala) and R6G peptide [(Arg)_6_-Gly]. PMOs for *rpsJ* (ribosomal protein) and *acpP* (fatty acid synthesis protein) were conjugated to the 3’ end of each peptide. Minimum inhibitory concentration (MIC) assays were performed in unique lab and clinical (including MDR) isolates using MOPS medium. Strain PAO1 biofilms were grown in MBEC plates for 24 hours, with PPMOs then dosed every 8 hours for 24 hours, followed by colony enumeration. For *in vivo* studies, mice were infected with strain PA103 intratracheally and given intranasal PPMO at 6 hours post-infection. Lung burden was determined at 24 hours post-infection.

**Results:**

MIC values of D-R6G-RpsJ and D-R6G-AcpP were comparable to their L-isomers, but D-RXR-RpsJ showed an improved MIC_90_ of 0.5 μM compared to the L-RXR-RpsJ MIC_90_ of 16 μM (Table 1). Both isomers demonstrated reduction of 48-hour biofilm to various degrees. D-PPMO biofilm reduction ranged from 1.5 logs to > 3 logs (D-R6G-AcpP and D-RXR-RpsJ, respectively). L-PPMO biofilm reduction ranged from 2 logs to > 4 logs. D-PPMOs were superior to their L-PPMO counterparts in our delayed *in vivo* acute pneumonia model, with most D-PPMOs resulting in an additional 50% decrease in lung burden.

**Figure:**
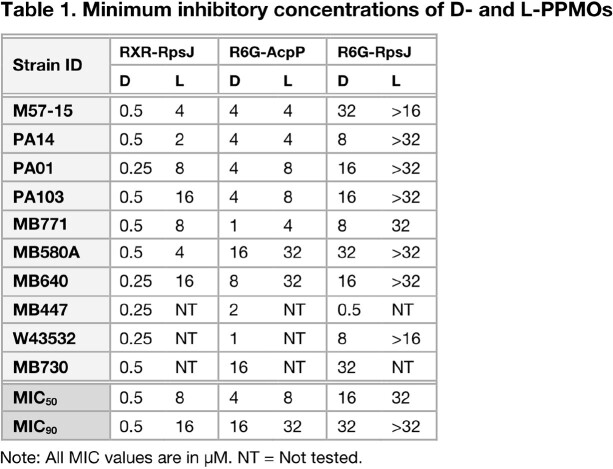

**Conclusion:**

D-PPMOs show similar or improved activity *in vitro* and *in vivo* when compared to L-PPMOs at the same dosage. Future studies will investigate the pharmacology of D-PPMOs, as they may represent a more biologically robust alternative to L-PPMOs for PA infections.

**Disclosures:**

**David E. Greenberg, MD**, University of Texas Southwestern Medical Center: Dr. Greenberg has numerous patents on PPMOs

